# Breast cancer and cervical cancer prevention programmes carried out by local government units in Poland in 2009–2014

**DOI:** 10.18632/oncotarget.24513

**Published:** 2018-04-24

**Authors:** Anna Augustynowicz, Aleksandra Czerw, Mariola Borowska, Adam Fronczak, Andrzej Deptała

**Affiliations:** ^1^ Department of Public Health, Medical University of Warsaw, Warsaw, Poland; ^2^ Department of Organization, Health Economics and Hospital Management, National Institute of Public Health - National Institute of Hygiene, Warsaw, Poland; ^3^ Department of Cancer Prevention, Medical University of Warsaw, Warsaw, Poland

**Keywords:** health policy programmes, local government unit, breast caner, cervical cancer

## Abstract

**Background:**

In 2014 the standardised incidence rate for breast cancer in Poland reached 51.6/100,000, while the mortality rate reached 14.8/100,000. The incidence rate for breast cancer in the EU was 106.6/100,000, the mortality rate – 22.4/100,000. In 2014 the incidence rate for cervical cancer in Poland was 8.8/100,000, the mortality rate – 4.5/100,000. The incidence rate in the EU was 11.3/100,000 and the mortality rate – 3.7/100,000.

**Objective:**

The aim of the paper was to establish the number of health policy programmes concerned with breast cancer and cervical cancer in women carried out in 2009–2014 by local government units, with specification of the type of programme, type of local government units that carried out the programmes and the costs of implementation of the programmes.

**Methods:**

The study was based on a desk research. The analysis covered data included in annual reports submitted by voivodes to Minister of Health, concerning health policy programmes implemented by local government units in 2009–2014.

**Results:**

The greatest number of programmes concerned with prevention of breast cancer and cervical cancer were implemented in municipalities, followed by counties and finally – self-governed voivodeships. The number of programmes concerned with primary prevention was three times smaller (656) than the number of programmes concerned with secondary prevention (2,229). The greatest number of primary prevention programmes were implemented in Dolnośląskie, Wielkopolskie and Mazowieckie Voivodeships, and the greatest number of secondary prevention programmes – in Wielkopolskie, Mazowieckie and Zachodniopomorskie Voivodeships.

**Conclusion:**

It was found that the number of programmes implemented by particular local government units and the financial resources employed in the implementation of the programmes were different. It is probable that some of the initiatives of local government units related to secondary prevention coincide with the actions undertaken under the National Programme for Fighting Cancer. The entities that carry out breast cancer and cervical cancer prevention programmes need to coordinate their actions.

## INTRODUCTION

Cancer is one the greatest health hazards in Poland. According to epidemiological estimates, the number of diagnosed patients and deaths due to cancer in the world and in Poland alike will probably continue to grow in the next decades, primarily among patients aged over 65 years [1]. There clearly is a need to undertake and continue actions aimed at preventing this public health problem in all its aspects. Preventive healthcare and health promotion should play a major role, especially in areas where they can have a particular impact on the incidence rates, e.g. in the case of breast cancer and cervical cancer.

In 2009–2014 in Poland breast cancer was the most common type of cancer diagnosed in women and the second most common cause of death of women [2]. In 2014 the standardised incidence rate for breast cancer was 51.6/100,000 [2]. In the EU the rate was 106.6/100,000 [3]. In 2014 in Poland the standardised mortality rate for breast cancer was 14.8/100,000 [1]. In the EU the rate was 22.4/100,000 [3]. The incidence of breast cancer is expected to grow. It is expected that by 2025 it will reach around 61/100,000 [4]. Based on the mortality trends, it is expected that the mortality rate will remain stable at the current level [4].

It should be noted that the incidence of cervical cancer in Poland has been decreasing. The number of deaths due to the disease has also been decreasing. In 2014 the standardised incidence rate for cervical cancer was 8.8/100,000 [2]. The standardised mortality rate was 4.5/100,000 [2]. The incidence rate in the EU was 11.3/100,000, the mortality rate – 3.7/100,000 [3]. If the present trends continue, it should be expected that by 2025 the incidence rate of cervical cancer will decrease to around 7–8/100,000. The mortality rate is also expected to drop to around 4/100,000 in 2025 [4].

The fight against cancer is one of the primary tasks of public health. High incidence and mortality rates among middle-aged women diagnosed with breast cancer are only two of the challenges faced by cancer prevention and treatment in Poland. Another serious problem is the low percentage of early diagnoses of cancer, including breast cancer and cervical cancer [5]. In Poland organised initiatives aimed at fighting cancer, including cervical cancer and breast cancer, are undertaken as part of the National Programme for Fighting Cancer [6, 7] and through health policy programmes that may be carried out by local government units, i.e. municipalities, counties and voivodeships. The programmes are prepared and implemented voluntarily. They may involve preventive and therapeutic actions [8].

In recent years the activity of local government units in the area of primary and secondary prevention of breast cancer and cervical cancer has not been a subject of any research. Reference literature includes studies related to programmes of 2006 and 2008 aimed at fighting cancer, caries and periodontal disease prevention programmes and programmes aimed at fighting cardiovascular diseases [9–11].

## RESULTS

In the period under review, local government units of all ranks implemented a total of 2,885 programmes. The programmes completed included breast cancer prevention programmes (1,646) and cervical cancer prevention programmes (1,187). Some of the programmes were designed as consolidated programmes and covered both cervical cancer and breast cancer (53). The programmes were addressed to women and girls of all ages. Breast cancer prevention consisted in breast self-exam training, mammography for women aged 50 to 69 years or ultrasound tests for women aged 25 to 35 years. Cervical cancer prevention consisted in administration of HPV vaccines and cervical screening tests. The financing also covered communications in the form of posters, announcements in the press, leaflets, videos.

The analysis covered the number of primary and secondary prevention programmes completed in particular voivodeships (Table 1).

**Table 1:** The number of primary and secondary prevention programmes completed in particular voivodeships and in total in the country

Voivodeship	Prevention			
	primary		secondary	
	*n*	%	*n*	%
dolnośląskie	89	39,6	136	60,4
kujawsko-pomorskie	20	54,1	17	45,9
lubelskie	33	32,0	70	68,0
łódzkie	47	40,2	70	59,8
małopolskie	40	26,1	113	73,9
mazowieckie	68	15,6	369	84,4
opolskie	47	56,0	37	44,0
podkarpackie	7	7,1	92	92,9
podlaskie	25	52,1	23	47,9
pomorskie	56	31,8	120	68,2
śląskie	34	23,8	109	76,2
świętokrzyskie	14	6,8	193	93,2
warmińsko-mazurskie	26	36,6	45	63,4
wielkopolskie	70	10,7	585	89,3
zachodniopomorskie	37	15,4	204	84,6
lubuskie	43	48,3	46	51,7
In total	656	22,7	2229	77,3

In Dolnośląskie, Lubelskie, Łódzkie, Małopolskie, Mazowieckie, Podkarpackie, Pomorskie, Śląskie, Świętokrzyskie, Warmińsko-Mazurskie, Wielkopolskie and Zachodniopomorskie Voivodeships there were more secondary prevention programmes than primary prevention programmes. In terms of results obtained for the entire country, secondary prevention programmes also prevailed over the other type of programmes. In Kujawsko-Pomorskie, Opolskie and Podlaskie Voivodeships there were more primary prevention programmes than secondary prevention programmes.

The greatest number of programmes were completed by municipalities (2,139), followed by counties (700) and self-governed voivodeships (46).

A vast majority of municipalities and counties did not run any primary and secondary prevention programmes associated with breast cancer and cervical cancer in the analysed period. Tables 2 and 3 present the number of programmes implemented by particular local government units (Tables 2 and 3).

**Table 2:** The number of primary and secondary prevention programmes concerned with breast cancer implemented by particular local government units in 2009–2014

Local government unit	No. of programmes	No. of units implementing programmes in particular years					
		2009	2010	2011	2012	2013	2014
Municipality	0	2392	2365	2373	2355	2364	2382
	1	40	64	55	69	47	44
	2	26	24	17	16	19	13
	3	12	11	16	8	11	7
	4	9	15	18	31	30	33
County	0	314	326	334	347	348	335
	1	41	38	38	22	24	32
	2	15	12	4	10	6	10
	3	8	3	3	1	1	2
	4	2	1	1	0	1	1
Voivodeship	0	10	14	12	14	14	14
	1	5	2	2	1	2	1
	2	1	0	1	1	0	1
	3	0	0	1	0	0	0

**Table 3:** The number of primary and secondary prevention programmes concerned with cervical cancer implemented by particular local government units in 2009–2014

Local government unit	No. of programmes	No. of units implementing programmes in particular years					
		2009	2010	2011	2012	2013	2014
Municipality	0	2412	2390	2396	2371	2387	2399
	1	38	62	48	72	53	42
	2	20	19	20	18	24	23
	3	8	7	12	6	3	2
	4	1	1	3	12	12	13
County	0	326	321	347	347	333	331
	1	38	47	24	27	35	36
	2	11	11	6	5	10	6
	3	3	0	2	1	2	3
	4	2	1	1	0	0	4
Voivodeship	0	14	11	12	13	10	11
	1	2	5	4	3	6	5
	2	0	0	0	0	0	0
	3	0	0	0	0	0	0

The analyses covered total costs of implementation of the programmes in PLN for municipalities, counties and voivodeships, total costs of programmes concerned with breast cancer and costs of programmes concerned with cervical cancer as well as the total cost of primary prevention and secondary prevention programmes.

The average total cost of programmes implemented by local government units in 2009–2014 was PLN 29,122.81 for municipalities, PLN 69,532.53 for counties and PLN 350,892.80 for voivodeships. As the highest-ranking local government units, voivodeships expended the largest amounts of money.

The average costs of breast cancer programmes, cervical cancer programmes and combined breast cancer and cervical cancer programmes reached comparable values. The average total cost of breast cancer prevention programmes was PLN 59,597.92. Similarly, the average total cost of combined programmes was PLN 62,551.59. Cervical cancer prevention programmes entailed the lowest average total costs - PLN 50,615.80.

After a review of the costs of primary and secondary prevention, it was established that the average total costs of primary prevention programmes were higher compared against the average total costs of secondary prevention. In 2009–2014 the average total cost of primary prevention programmes was PLN 67,933.87. The average total cost of secondary prevention programmes was PLN 45,489.37.

The analysis also looked at the programmes that involved primary and secondary prevention actions. The greatest number of primary prevention programmes were completed by municipalities (418), followed by counties (229) and self-governed voivodeships (8). Most of the programmes concerned cervical cancer (556), with fewer programmes concerned with breast cancer (85) or both cancer types (15).

The greatest number of secondary prevention programmes were completed by municipalities (1,719), followed by counties (471) and self-governed voivodeships (38). Most of the programmes concerned breast cancer (1,561), with fewer programmes concerned with cervical cancer (630) or both cancer types (38).

## DISCUSSION

The purpose of the analysis was to establish the number of primary and secondary prevention programmes concerned with breast cancer and cervical cancer carried out by particular local government units. Local government units of all ranks engaged in such actions. Further, there is the National Programme for Fighting Cancer in place in the entire country, which includes the Population Programme for Prevention and Early Detection of Cervical Cancer and Population Programme for Early Detection of Breast Cancer. The programmes carried out throughout the country include cervical screening tests and mammography. Hence, it is probable that identical actions are carried out within one area. This might serve as evidence of insufficient coordination, lack of cooperation or exchange of information between entities that carry out the programmes. With regard to this problem, local government units should be advised to undertake initiatives that complement actions pursued on a national scale, e.g. promote screening tests. This is especially important in voivodeships where mammography and cervical screening tests were rarely performed. There is also a need for increased coordination of actions undertaken by local governments and government administration to avoid duplicating actions and, in effect, improve the rationality in public spending. The Supreme Audit Office came to the same conclusion during an audit whose purpose was to evaluate the efficiency of local government units in implementing health policy programmes [12].

It was found that the number of programmes implemented by particular local government units and the financial resources employed in the implementation of the programmes were different. With regard to the number of programmes per a given type of local government unit, municipalities proved to be the least efficient. In 2009–2014 one municipality completed on average one primary and secondary prevention programme concerned with breast cancer and cervical cancer. Municipalities also expended the smallest amount of money on the programmes. The analysis of the expenditure structure in municipalities shows that they expended the funds, first and foremost, on fulfilment of their statutory obligations. This might serve as evidence of unequal access to health policy programmes. In consequence, access to a programme could depend on the place of residence and be limited to women resident in municipalities with low income. This conclusion coincides with the conclusions formulated by the Supreme Audit Office [12].

Another aspect evaluated as part of assessment of the activity of local government units was the number of primary prevention programmes. In total, local government units completed 556 primary prevention programmes concerned with cervical cancer that involved education, promoting healthy habits and administration of HPV vaccines. HPV vaccines were given to 150,850 girls. There were over 6 times more primary prevention programmes concerned with cervical cancer than programmes concerned with breast cancer. The difference is probably a consequence of the fact that primary prevention is crucial in reducing the incidence of cervical cancer [13]. Another important factor could have been the guidelines of WHO [14] and recommendations of international and national learned societies saying that primary prevention, especially HPV vaccines, should complement regular cervical screening tests [13, 15, 16].

It was found that the level of engagement of local government units in implementation of secondary prevention programmes was different in particular voivodeships. The difference should be a consequence of identified health needs and health condition of residents. In 2009 standardised mortality rates for breast cancer reached the highest values in Kujawsko-Pomorskie Voivodeship (17.1/100,000), Wielkopolskie and Śląskie Voivodeships (15.5/100,000), Łódzkie Voivodeship (14.9/100,000) and Warmińsko-Mazurskie Voivodeship (14.3/100,000) [17]. The conclusion from the analysis of the above data is that Wielkopolskie, Śląskie and Mazowieckie Voivodeships addressed women’s health needs associated with breast cancer most efficiently by implementing in 2009–2014 the highest number of secondary prevention programmes. Unlike Kujawsko-Pomorskie and Warmińsko-Mazurskie Voivodeships, which did not implement a sufficient number of programmes. In 2009 the highest mortality rates for cervical cancer were recorded in Warmińsko-Mazurskie Voivodeship (7.1/100,000), Wielkopolskie Voivodeship (6.4/100,000) and Pomorskie Voivodeship (6.2/100,000). The rates were also high in Lubuskie (5.8/100,000), Mazowieckie, Łódzkie and Kujawsko-Pomorskie (5.4/100,000) Voivodeships [17]. A positive trend can be noticed in Wielkopolskie, Pomorskie and Mazowieckie Voivodeships – the higher the mortality rate, the higher the number of implemented programmes. No such trend was noted in Kujawsko-Pomorskie, Warmińsko-Mazurskie and Łódzkie Voivodeships. This means that at least some of the local government units did not factor in the health needs and health condition of residents in the process of making decisions about development, financing and implementation of health policy programmes. This is contrary to Art. 7 Sec. 1 Clause 1, Art. 8 Clause 1 and Art. 9 Clause 1 of the Act on healthcare services financed with public funds.

The information included in the reports does not allow for evaluation of the efficiency of health policy programmes and the efficiency of actions undertaken as part of such programmes. According to the Act on healthcare services financed with public funds, local government units are obligated to evaluate the effects of implemented programmes. The reports lack information about the number of people involved in particular programmes. Local government units also failed to specify the number of people diagnosed with a given disease.

The example of Europe shows that systemic solutions aimed at combating cancers should be contingent on the type of cancer. The battle against breast cancer should involve screening programmes, which enable early detection and, thereafter, coordinated diagnosis of suspicious lesions [18]. In Finland and Sweden high detectability of breast cancer and small incidence of cervical cancer are a result of successful population screening programmes [19]. Another finding was that the efficiency of cancer treatment, including especially treatment of breast cancer, was contingent on the effects and time of launching of national programmes for early detection of cancer [20]. In Finland the mammography programme was launched in 1986 and it has reached over 80% of patients. In Sweden the mammography programme was launched at the beginning of the 1970 s. In 1997 it was awarded the status of a national programme [21]. The initial controversy surrounding the efficiency of screening tests in reducing mortality due to breast cancer [22] was eliminated after years-long observation [23].

## MATERIALS AND METHODS

The study was based on a desk research. The analysis covered data included in annual reports formulated by voivodes and submitted to Minister of Health concerning all health policy programmes implemented by local government units. The analysis covered all programmes whose name, objective or description of tasks indicated that they concern actions related to primary or secondary prevention of breast cancer and cervical cancer in women. Primary prevention programmes included programmes that aimed to decrease the incidence of cancer and impact of risk factors and programmes concerned with health education within the society. Secondary prevention programmes included screening test programmes.

The analysis covered programmes implemented by local government units of all ranks, i.e. by the largest administrative units (voivodeships), by second-degree administrative units that rank below voivodeships (counties) and by primary administrative units (municipalities). Counties included also county towns.

The analyses covered the number of completed primary and secondary prevention programmes in total, depending on the local government unit and cancer type, and the number of programmes completed by all the voivodeships and in the country in total. They also covered the costs of implementation of the programmes depending on the local government unit, cancer type and type of the programme. There was a separate analysis of primary and secondary prevention programmes depending on the local government unit and cancer type and of the costs of implementation depending on the type of local government unit and cancer type.

## CONCLUSIONS

It was found that the number of programmes implemented by particular local government units and the financial resources employed in the implementation of the programmes were different.

It is probable that some of the initiatives of local government units related to secondary prevention of breast cancer and cervical cancer coincide with the actions undertaken under the National Programme for Fighting Cancer. We would need to continue research to confirm this.

The entities that carry out breast cancer and cervical cancer prevention programmes need to coordinate their actions.

Local government units should be subject to research to specify the determinants of development and implementation of health policy programmes and to present the outcomes of the implemented programmes.
